# Supplementary material for: Causal Relationship between Aging and Anorexia Nervosa: A White-Matter-Microstructure-Mediated Mendelian Randomization Analysis
Source: Biomedicines. 2024 Aug 16;12(8):1874. doi: 10.3390/biomedicines12081874 (PMC11351342; doi:10.3390/biomedicines12081874)
Supplement: Supplementary file 1 [file biomedicines-12-01874-s001.zip › Supplementary Figure/Figure S4/Singe SNP plot of MR analysis for TL to ubm-b-1764.pdf]

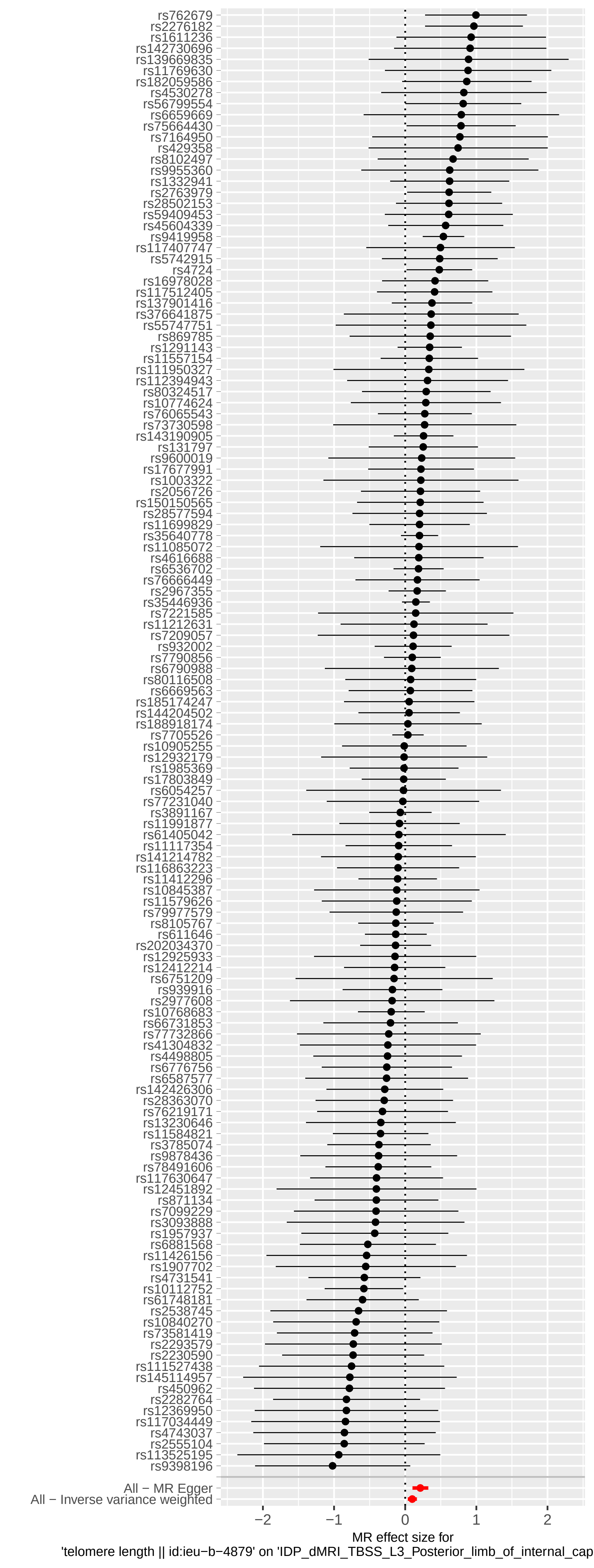
rs762679
rs2276182
rs1611236
rs142730696
rs139669835
rs11769630
rs182059586
rs4530278
rs56799554
rs6659669
rs75664430
rs7164950
rs429358
rs8102497
rs9955360
rs1332941
rs2763979
rs28502153
rs59409453
rs45604339
rs9419958
rs117407747
rs5742915
rs4724
rs16978028
rs117512405
rs137901416
rs376641875
rs55747751
rs869785
rs1291143
rs11557154
rs111950327
rs112394943
rs80324517
rs10774624
rs76065543
rs73730598
rs143190905
rs131797
rs9600019
rs17677991
rs1003322
rs2056726
rs150150565
rs28577594
rs11699829
rs35640778
rs11085072
rs4616688
rs6536702
rs76666449
rs2967355
rs35446936
rs7221585
rs11212631
rs7209057
rs932002
rs7790856
rs6790988
rs80116508
rs6669563
rs185174247
rs144204502
rs188918174
rs7705526
rs10905255
rs12932179
rs1985369
rs17803849
rs6054257
rs77231040
rs3891167
rs11991877
rs61405042
rs11117354
rs141214782
rs116863223
rs11412296
rs10845387
rs11579626
rs79977579
rs8105767
rs611646
rs202034370
rs12925933
rs12412214
rs6751209
rs939916
rs2977608
rs10768683
rs66731853
rs77732866
rs41304832
rs4498805
rs6776756
rs6587577
rs142426306
rs28363070
rs76219171
rs13230646
rs11584821
rs3785074
rs9878436
rs78491606
rs117630647
rs12451892
rs871134
rs7099229
rs3093888
rs1957937
rs6881568
rs11426156
rs1907702
rs4731541
rs10112752
rs61748181
rs2538745
rs10840270
rs73581419
rs2293579
rs2230590
rs111527438
rs145114957
rs450962
rs2282764
rs12369950
rs117034449
rs4743037
rs2555104
rs113525195
rs9398196
All − MR Egger
All − Inverse variance weighted
−2
−1
0
1
2
MR effect size for
'telomere length || id:ieu−b−4879' on 'IDP_dMRI_TBSS_L3_Posterior_limb_of_internal_cap
